# Medical Teaching in the Fifteenth Century

**Published:** 1903-10-03

**Authors:** 


### Medical Teaching in the Fifteenth Century.

Professor Clifford Allbutt recently contributed an article to *The Medical Chronicle* dealing with Professor Ferrari da Grado, who occupied the chair of medicine in the University of Pavia from 1432 to 1472, in which he gives many details about the state of the universities in the fifteenth century. He points out that the rivalry of the new universities of the middle ages was quite as acute as it is at the present day in the north of England. Thus the University of Toulouse issued the following advertisement :—"This is the promised land where flows milk and honey, where rich pastures are green and trees are laden with fruit: the air is finer than in any other province, and at Toulouse wine, bread, meat and fish are cheap." The University of Pavia was not behind in proclaiming its many advantages—cheap lodgings, food almost for nothing, and the best professors in Italy. Bologna, Padua, Montpellier, and the rest boasted likewise of the beauty of their country, the salubrity of their climate, the accessibility of the town, and the advantages of their school. The Universities were sometimes hard pressed for money, as is evident from the following letter written by the Rector of the Jurists at the University of Pavia. It is addressed to the Duke of Milan, and is dated October 27th, 1447 :—"Very Illustrious Prince and Very dear Master,—Of late, in order to pay salaries in arrears, we have had often and importunately to address ourselves to the Jew, who has been very useful to us; but now, forsooth, the Jew will lend no more, and hence the University and its studies are in grievous straits. As this Hebrew is causing great injury to our University, we have decided to appeal to your Excellency to take steps to compel the Hebrew to make us loans as before." This letter may be compared with one written by Gilbert Kymer, M.D., Chancellor of the University of Oxford, which is dated 1453 and is directed to the Master of St. Thomas's Hospital in London. The University by the hand of Dr. Gilbert Kymer "confidently begs that you will intercede for us with the wealthy citizens of London that they may assist us in building the new schools (the present divinity school), and that you will advise our Chancellor how to cast his net on the right side of the ship when he appeals to them for assistance." The students at a university were drawn from a very different class to those of the present day. Those who were poor were often in the most dire straits, and a poor student of the fifteenth century says: "The time I should spend at lectures and in study I am driven to waste in begging from door to door, crying scores and scores of times, 'Charity, charity, dear masters,' and getting the answer, 'Begone, and God be with you.' I appeal both to ecclesiastics and laymen, and am mostly driven from the door, or perchance someone may say, 'Wait a bit,' when I may get a dirty scrap of bread which a dog would reject, or I may get fusty beans, bits of skin or gristle, or sour wine. By night I traverse the city, in one hand a stick to defend me against the dogs, in the other a bag to gather remnants of bread, fish, or vegetables, and a water gourd. Often I have to return home, filthy with mud, to try to appease my barking stomach with any scraps I might have picked up." It is clear that the poverty of English University students lasted till late in the reign of Queen Elizabeth, because the second Poor Law passed in 1572 classed as a vagabond "scholars of the Universities begging without license from the University authorities." To relieve such wretchedness it was that colleges were founded and were ruled on the monastic system. But if the poor were very poor the rich were very rich, and the ceremony of graduation might be carried out at enormous expense. We read in the notes of the two medical students, Felix and Thomas Platter, of the graduation ceremonies at Basel for which the hall of the University was insufficient. The whole faculty gathers in the Cathedral, whither the candidate fares on horseback, preceded by trumpeters. The previous night he had serenaded all the doctors, surgeons, and apothecaries with a band of trumpets, fifes, and stringed instruments. In the hall a collation is served at the candidate's expense, "de optimis confectionibus et vino dalmatico." As the procession passes through the town, the merchants shut up their shops and the artisans stop their work. Upon the high platform of the Cathedral the Chancellor pronounces a discourse in Latin and hands to the candidate a book, first open and then closed, caps him, pronounces certain ritual words, puts a gold ring upon his finger, and finally gives him the kiss of peace. A banquet to the doctors, students, officers, and notables of the city followed this ceremony, when the graduate was wont to return thanks in five or six languages. A similar feast took place in England when a man was made a serjeant-at-law, and until recently at Oxford a student might elect to pay special fees and graduate as a "grand compounder." All this was not done for nothing, the examination cost about 600 livres, and the conventus 80: the Chancellor received 25, and he or his vicar about 6 more. Besides these fixed fees, the candidate had to defray the cost of the banquet and of the dresses for a great many people, just as the serjeant, when he was admitted to the Order of the Coif, had to present gold rings to the Sovereign, the Lord Chancellor, and others, *fidei symbolo*. As early as 1311, these expenses had become so great that the Pope ordered that candidates should swear not to spend more than 500 livres over and above the fixed fees of graduation. In the Italian Universities at this time there were three degrees—the Bachelor, the Licentiate, and the Doctor, the latter being sometimes replaced by the Master. The Bachelorship was rather a certificate of study than a degree. There was no examination, but certain certificates were produced, and other conditions were required. But at Montpellier an Act was required for M.B., and the Act was a serious business, lasting from early morning till late at night. The Act for the Degree of M.D. consisted of a thesis followed by discussion, but of personal experience there was nothing, of observation nothing. The commentary of the schools was sufficient for all purposes, and the spirit of true criticism had ceased. The degree of Doctor was not necessary for the practice of medicine: it was the completion of the scientific study of medicine: a distinction between academic training and technical instruction too often lost sight of at the present day.

